# Promoting citizen science in the energy sector: Generation Solar, an open database of small-scale solar photovoltaic installations

**DOI:** 10.12688/openreseurope.13069.2

**Published:** 2021-05-21

**Authors:** Carlos del Cañizo, Ana Belén Cristóbal, Luisa Barbosa, Gema Revuelta, Sabine Haas, Marta Victoria, Martin Brocklehurst

**Affiliations:** 1 Instituto de Energía Solar, Universidad Politécnica de Madrid, Madrid, Spain; 2 Centro de Estudios de Ciencia, Comunicación y Sociedad, Universitat Pompeu Fabra, Barcelona, Spain; 3 Reiner Lemoine Institut, Berlin, Germany; 4 Department of Engineering, Aarhus University, Aarhus, Denmark; 5 KempleyGreen Consultants, Gloucestershire, UK

**Keywords:** Citizen Science, Public Engagement, Energy, Photovoltaics, Open Data

## Abstract

Citizen science is becoming an effective approach in building a new relationship between science and society, in which the desire of citizens to participate actively in knowledge production meets the needs of researchers. A citizen science initiative dealing with the development of photovoltaics (PV) is presented. To generate a “responsible” initiative, the research question has been designed collectively from the beginning, involving diverse actors in order to encourage creativity while addressing their interests and concerns. The result has been called Generation Solar. It aims at co-creating an open database of PV installations including their technical characteristics, and an online map for visualizing them. The initiative responds to a clear scientific demand; an important drawback for researchers working on energy modelling and predictions of production lays precisely in the lack of information about these installations’ locations and characteristics. The initiative invites citizens, companies and public institutions with a PV installation to collaborate by providing such data. Data will follow the format of Open Power System Data in order to be fully exploitable by the scientific community and society. The success of the initiative will rely on the capacity to mobilize citizens and register the largest possible number of installations worldwide.

## 1 Introduction

The achievement of the Sustainable Development Goals (SDGs) established by the international community as part of the 2030 Agenda will only be possible with the determined commitment of society as a whole (governments, companies, academy and civil society), and needs the deployment of all available tools, with science and technology among them ^1^. Research and innovation should focus on these challenges, and important efforts have been made in the last few years to base this demand for alignment with societal goals on solid principles, giving birth to the concept of responsible research and innovation (RRI) ^2^. RRI implies introducing changes in the way research and development is conducted, in particular by engaging all stakeholders in the entire research and innovation cycle, and not only in the evaluation of results.

In this context, citizen science emerges as a powerful tool to match the potential benefits that research can bring to the needs, values and aspirations of citizens. It is a rapidly growing field, with an explosion of related activities and experiences in a wide range of science disciplines ^3^, such as astronomy, biodiversity, medicine, metrology and transport.

Efforts have been made to incorporate this kind of practices in the energy sector, covering aspects such as the evaluation of transition scenarios ^4^, the engagement in energy efficiency and energy demand management ^5, 6^, the support in the performance assessment of photovoltaic systems ^7^ and the mapping of existing PV installations into OpenStreetMap ^8^. It is clear that the urgent energy transition in which we are immersed, with the relevance of renewable energies, will only be possible through the concerted action of all stakeholders, putting into practice the benefits of a “quadruple helix of innovation” ^9– 11^, and going beyond social acceptance by reinforcing the participation in decision-making processes ^12, 13^. 

This paper presents a citizen science initiative in the photovoltaic (PV) sector, that intends to build an open database of PV installations that can give input, among others, to researchers working on energy modelling and predictions of production from PV plants.

Firstly, in section 2 we highlight the role that citizen science is beginning to play in the European Research Agenda, and how our initiative aligns with it. Then, in section 3 we describe the engagement process that has led to this specific initiative, as it was carefully designed to make sure that it responds to a real need in the research field, and at the same time meets the expectations of citizens. Section 4 describes in detail the initiative Generation Solar, implemented as an app.

## 2 Citizen science as part of the European research agenda

### 2.1 Matching science achievements and citizens’ aspirations

Citizen science refers to the “ *general public engagement in scientific research activities when citizens actively contribute to science either with their intellectual effort or surrounding knowledge or with their tools and resources*” ^14^. The concept of citizen science emerged and developed in the second half of the 20 ^th^ century, but it has been in the 21 ^st^ century when its potential has exploded, as a consequence of the democratization of knowledge, the internet revolution and the move towards open science ^15^.

The European Commission has embraced the idea that opening the research and innovation system to the participation and collective intelligence of society can significantly contribute to its success in finding solutions to the challenges we face. In Horizon 2020 it has dedicated a relevant budget (462 million €) to citizen science and citizen engagement projects under the SwafS program (Science with and for Society), under the premise that “ *Citizen science can make science more socially relevant, accelerate and enable production of new scientific knowledge, increase public awareness about science and ownership of policy making, as well as increase the prevalence of evidence-based policy making*” ^16^. This approach is also present in some of the recent calls included in the energy-climate work programmes in Horizon 2020 and Horizon Europe, confirming its relevance for policy and decision makers. For example, the calls in Horizon 2020 - Work Programme 2018–2020 – Cross-cutting activities included in *Area 10: Empowering citizens for the transition towards a climate neutral, sustainable Europe*, in particular the call “LC-GD-10-3-2020: Enabling citizens to act on climate change, for sustainable development and environmental protection through education, citizen science, observation initiatives, and civic engagement”.

There is already enough evidence to be able to systematize the key principles that define citizen science ^17^, and it is clear that the benefits that citizen science brings can be very large, but important challenges need to be tackled: among them, the avoidance of ethical lapses and security risks, or the design and adoption of indicators to measure outcomes ^14, 15^.

On the other hand, it is probably unrealistic to expect that all researchers involved in projects related to societal challenges are going to include citizen science as normal practice within their research activities, because defining and launching such initiatives is quite time-consuming and effort-demanding, and raising a new community takes time, effort and money. It is clear that adopting citizen science implies taking academic-based research away from its “comfort zone”; among other things, it has to learn how to involve different stakeholders in the research process, it should define new incentives for interaction with citizens, and also design models for the long-term sustainability of citizen science projects. It should also adopt Open Science policies and provide appropriate tools ^12^, and lay out success stories that can inspire researchers ^15^.

GRECO (Fostering the next generation of a European photovoltaic society through Open Science), a SwafS project funded by Horizon 2020, wants to put these principles of RRI and public engagement into practice in a research project in the PV field, studying the conditions in which they can improve the quality of the research and strengthen the social acceptance of the PV products under development ^18^.

### 2.2 Public engagement in a research project

The GRECO project addresses several research lines in the PV field at different technologies readiness levels (TRLs), with the goal of demonstrating that public funded science can be performed in a more responsible way. This is being tested in a research line related to the ageing and repairing of PV modules, in a second one devoted to PV solutions for irrigation, and in a third one related to the development of next generation PV concepts, which includes novel tandem solar cells, micro-concentration PV systems and PV heat pumps.

 From GRECO’s experience, we can identify three lines for the involvement of citizens which can have high impact: public engagement through mobilization and mutual learning actions, through open innovation processes, and through the direct collaboration of citizens. They are described in more detail in 19. Here we will focus in one of the experiences of the latter case, the Citizen Science initiative.

## 3 Development of a responsible citizen science initiative

Promoting a citizen science initiative for a research project, without taking into account citizens in its design, is somewhat contradictory. To avoid that, we aimed at the development of a “responsible” citizen science initiative, in the sense it respects the RRI principles and is the result of a co-creation process between citizens and scientists.

Even if it is particularized here for the PV sector, we think that our experience is hence relevant for the promotion of citizen science in a wide range of technological sectors.

The methodology that we have established to develop a responsible Citizen Science Initiative consists of the following four steps.

### 3.1 Step 1: input from professionals

In order to launch a citizen science initiative with real value for the advancement of professional science, academic researchers must be included in the design phase, with the double objective of identifying relevant research questions and raising awareness of the support that citizens can provide to address them. Otherwise, we take the risk of generating a movement of people collecting or analyzing data with the unique purpose of supporting science education, which is valid but not useful as input for a scientific project. This is especially valuable in engineering fields, less represented than biomedical and biological disciplines in public engagement actions.

For that, face-to-face brainstorming activities were carried out in several sessions in different countries to explore ideas from PV-related personnel around three questions, that were formulated to help the respondents think beyond their specific topics of expertise: How can citizens collaborate in our research? What are the main barriers towards the integration of PV in society? What does our research give in return to citizens? Approximately 60 researchers participated. Their input was complemented by an online survey that was distributed during two months among academic networks and other communities involved with solar energy and communication. Information about the data treatment was given for both the face-to-face meetings and for the online survey, and consent sheets collected.

A 9-page document summarizing all responses collected (around 100, see underlying data) was compiled and is openly available in Zenodo ^20^. It gathers a handful of ideas that were offered as a starting inspiring point to the participants in step 2.

### 3.2 Step 2: co-creation with society

**Figure 1.**
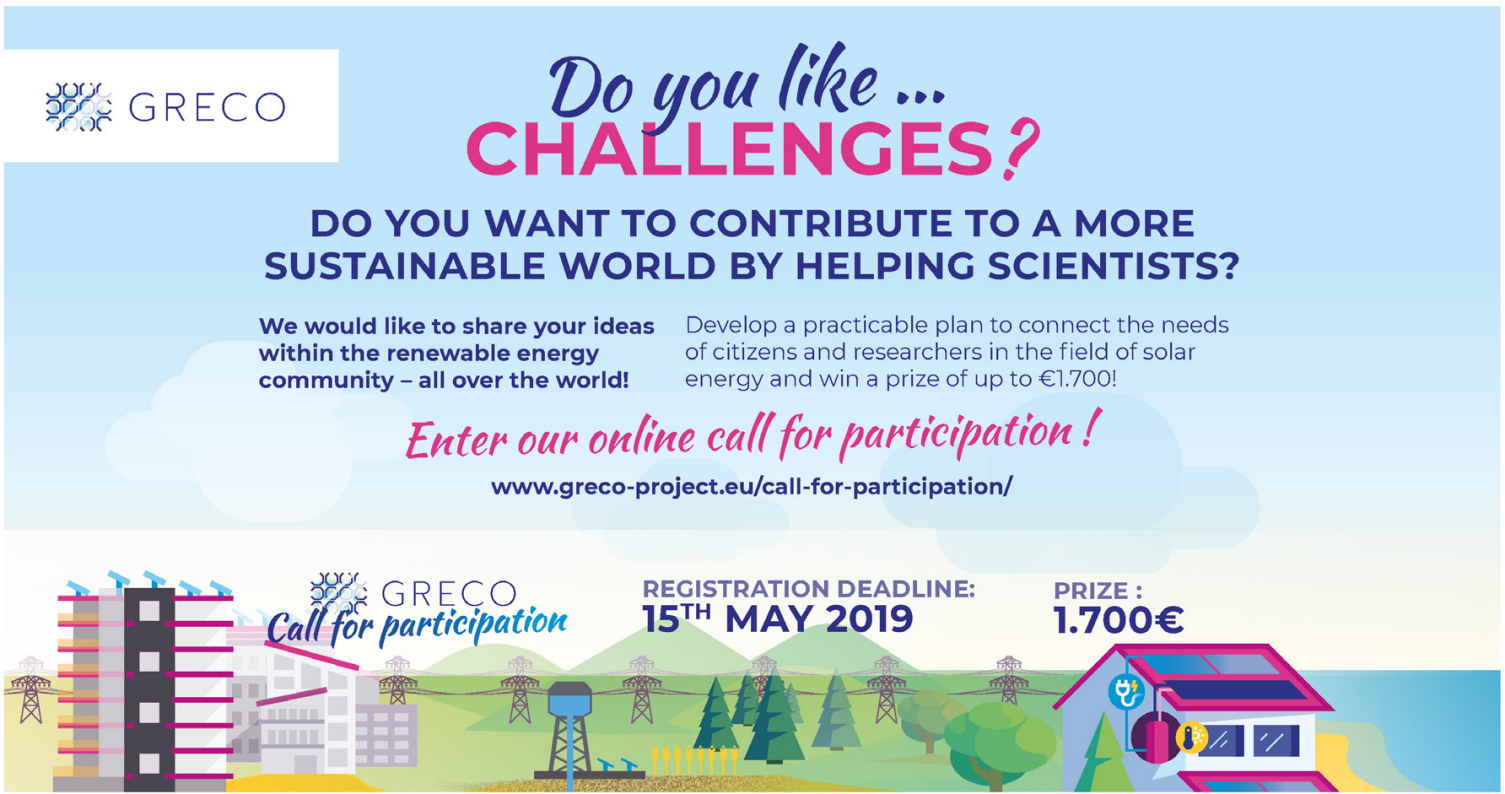
Call for ideas to design a PV citizen science initiative.

In this second phase, a call for participation was launched ( Figure 1), and disseminated with the support of flyers, a newsletter, press releases, and a videoclip, translated to several languages, throughout different communication channels: GRECO website and social networks, local newspapers and magazines, networks of the GRECO partners. An online contest, inspired in a hackathon ^21^, was designed to motivate participants to develop a working plan and submit it for evaluation. 

In a hackathon, participants are brought virtually together to engage in brief, intensive collaborative work to solve a challenge. Although hackathons started linked to the field of software or IT development, they have more and more been used in all disciplines and proven very powerful for knowledge exchange and broader problem framing ^22, 23^.

The contest finally gathered around 60 registered participants from 15 different countries, ran for an entire week and made use of the open source e-learning software Chamilo. Having as starting point the responses gathered in step 1, the participants, individually or in teams, had an entire week to propose an initiative in citizen science for the PV field, conveying a clear and feasible idea, together with the methodology and the tools to develop it.

### 3.3 Step 3: assessment and selection

After the contest had been closed, an assessment committee revised 12 proposals that represented the submission of 30 teams and individuals. Several criteria covering different aspects were established and tabulated (see Table 1), to support the selection of the winning proposal, that would be implemented within the GRECO project. Other prizes were established to reward specific traits as creativity and team-building. This served as recognition for brilliant ideas that deserved attention, even if they could not be developed by the GRECO project.

**Table 1.** Assessment criteria for the selection of the citizen science initiative.

Assessment criteria	Description
Mastering the challenge (20%)	The idea successfully responds to the challenge, by developing a working plan of a citizen science project for solar energy research.
Creativity (20%)	The proposal suggests original and unusual ideas, either by creating something new or by giving new uses to the existing tools.
Feasibility (20%)	The proposal can be developed by GRECO during the following six months and it considers responsibly the technical, economic, social and environmental aspects. Moreover, it can be implemented in the solar energy research community.
Methodological exactness (15%)	The working plan describes how to develop the proposal, including the target group (i.e., schools, universities, solar energy electricity users, etc.), resources, main tasks and timetable. Furthermore, if the proposal includes the development of specific software or device, their description is also included.
Global reproducibility (15%)	The proposal presents an idea that might be used across the globe and promotes international cooperation.
Clarity and visual presentation (10%)	The idea is presented in a clear, understandable and visually appealing way.

The highest score and hence the major prize was awarded to the proposal “Open database of rooftop solar PV installations”, which fully satisfied the characteristics demanded for GRECOs’ Citizen Science initiative (in particular, relevant citizen participation to respond to a relevant research question) while keeping its development within the framework of what the project could achieve. The winning proposal aimed at providing support to scientists that model future energy systems with high renewable penetration by creating a collaborative database under an open license regarding the main characteristics of PV installations around the world, fed with the information provided by citizens. In this way, it addressed some of the ideas coming from the survey in step 1 and summarized in 20: for example, “Identifying locations where solar energy could replace other sources”, “Providing their experience, especially those that have installed a PV system at home”, “Sharing data from their solar devices”, “Send data of photovoltaic installations”, “[get to know] how solar energy will fulfill the energy needs in their daily life”, etc.

### 3.4 Step 4: technical development

The technical implementation of the open database, named “Generation Solar”, was subcontracted to a company specialized in software development, and it was launched in April 2020.

The web version of the initiative is accessible at https://generationsolar.ies.upm.es, and apps are available for iOS and Android.

Special attention has been taken so that data follows the format and instructions from the Open Power System Data, in order to be fully exploitable by the scientific community and society ^24^. This will enable the maintenance of an open approach in energy modelling which is key to ensure transparency and reproducibility of the results ^25^. The details of Generation Solar are presented in the next section.

## 4 Generation Solar

When scientists model future energy systems with high renewable penetration, they need to know where the current installations are located, as well as make hypotheses on their future deployment. This information is not fully available now; although some databases for PV exist, they are private, include only information from very few countries ^7, 26, 27^, are outdated ^28^, only provide approximate locations, or lack important data such as the orientation and inclination of the modules. On the other side, citizens are willing to take part in the energy transition and small-scale rooftop PV allows them to become energy producers. Participating in the collaborative database of PV installations, Generation Solar can promote interest in, and acceptance of, PV. Compared to large power plants, the distributed nature of PV makes it more difficult to gather information regarding the installed capacity, the spatial distribution, and the configuration of the systems. Citizens can play a key role here, creating and updating a detailed database of rooftop PV installations that will be key to enhance the modelling and understanding of future distributed power systems. It may, for example, help answer research questions such as:

- Is the latitude determining the tilt of the panels, or the rooftop inclination? How is this evolving through time? (i.e., change of azimuth towards the West to match the consumption)
- How is the DC/AC ratio sized, and how is this changing with time?
- How are batteries being introduced in these types of installations?
- Can the information in Generation Solar complement, or help to find inconsistencies with, the current information available in some countries on registered PV installations?
- Can it help to contrast with the data gathered by approaches based on satellite images?
- What are the differences in type of ownership (rooftop vs share in large plants)? How is this influenced by country or region?

In this respect, Generation Solar can contribute to give support and continuation to works such as those of ^29, 30^, guaranteeing an open an easy access to its database. 

### 4.1 The app

The citizens introduce data from the installation that they are familiar with (location, latitude, longitude, orientation and inclination of the panels, technology used, commissioning date, etc.) and will keep this information updated, e.g., by indicating if the installation is upgraded to higher capacity or decommissioned.

**Figure 2.**
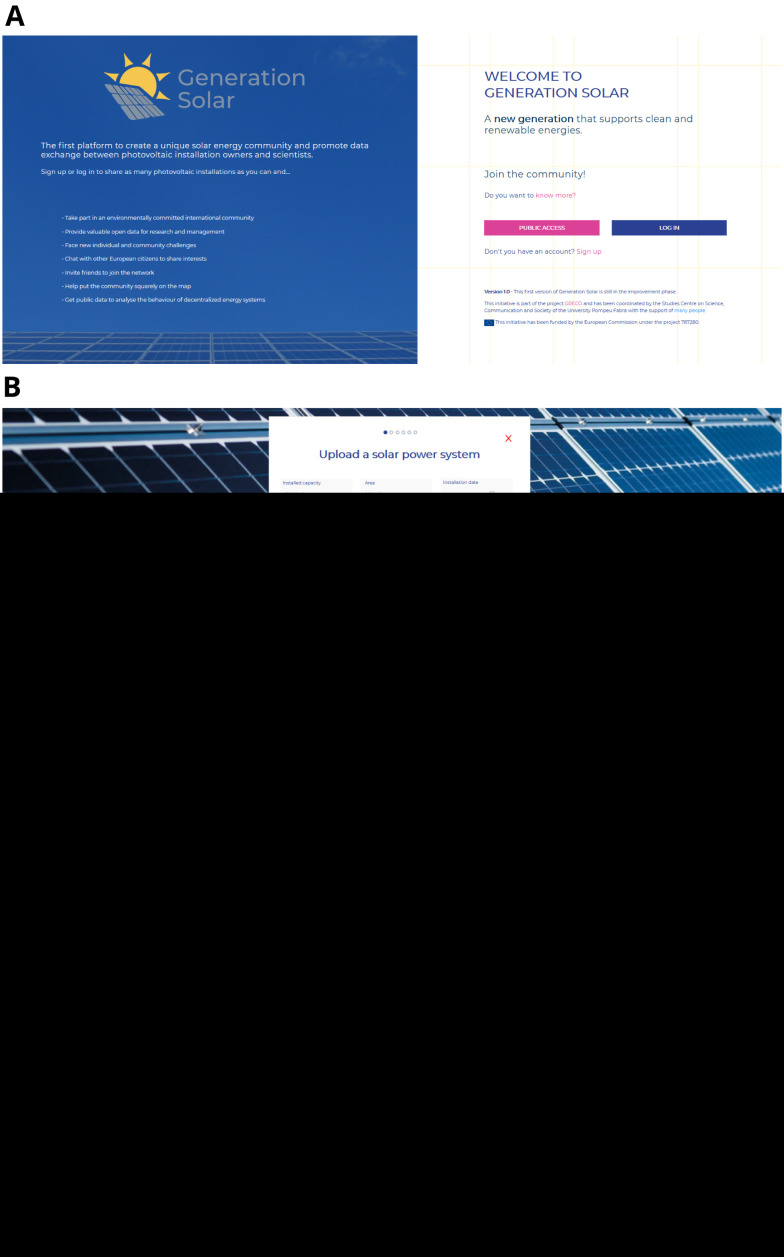
Some screenshots of Generation Solar app.

Screenshots are shown in Figure 2 as an example of the appearance of the web interface.

### 4.2 Registering a PV installation

In the landing page you may click on “public access”, giving access to a public map where you can find all the solar installations that have been registered, or “log-in” after signing up, to fully exploit Generation Solar’s possibilities.

Once logged in, you can explore the map with all the registered installations and their characteristics, chat with other users, check some global statistics, including a rough estimate of how much CO _2_ these installations are saving, and also access to some functionalities that have been added to motivate people: adding many installations and a lot of solar power, getting as many likes as possible, and engaging in debates with the community, or becoming an influencer by inviting others, aspects that have been demonstrated to be very relevant in many studies of social acceptance of the PV technology (see for example ^31– 33^).

The centrepiece of Generation Solar is the possibility to add installations. There you should fill in as much information on your solar power system as you can: installed capacity, module technology, inverter capacity, coordinates, orientation and tilt are the main ones. You can also upload some pictures of the installation, and give other details you consider relevant in a “observations” field.

That way, you will have your installation appearing on the global map.

### 4.3 Data output

Some basic statistics are shown, such as the total number of installations and their power, as a well as a rough estimation of the CO _2_ emissions saved by them.

The full Generation Solar database can be easily downloaded as csv file under a CC BY 4.0 license.

Note that in order to protect the privacy of the citizen scientists, the information about the installations is stored in a way that it has a spatial resolution of 1.1 km, to avoid the precise identification of their locations.

## 5 Conclusions

Generation Solar is a Citizen Science Initiative in the energy field that takes advantage of the participation of citizens to build an open database of distributed solar PV installations, to give input to researchers working on future energy systems simulating different energy supply scenarios and predicting production from PV plants. It will be a useful tool for research as long as it succeeds in bringing together the input from a very large number of citizens all over the world.

A web version of the app is accessible at https://generationsolar.ies.upm.es, and it can also be freely downloaded for iOS and Android devices.

There are already plans for upgrading the app to incorporate new functionalities, such as an estimation of the energy production of registered installations to benchmark the real production indicated by the electricity meter, and a more detailed calculation of the CO _2_ emission savings due to this PV production.

The value of Generation Solar is not restricted to the usefulness of the database itself, for which the engagement of citizens is crucial due to the decentralized nature of PV installations; it also comes from the design process, that may serve as a guide for the development of other Citizen Science initiatives. Conceived to build a “responsible” citizen science initiative, it gives emphasis to participatory and co-creation aspects, to make sure that the initiative responds to a real need in the research field, and at the same time meets the expectations of citizens.

The urgent changes that we need to implement in the way we consume and produce energy will only be possible with the commitment of the whole society, beginning with the citizens themselves, and Citizen Science is a powerful means to accomplish this engagement. It has already demonstrated its success in many scientific domains, and should also be promoted in the energy sector, and for that researchers and professionals need support, tools, resources, and successful stories to get inspiration from.

## Data availability

Zenodo: Underlying data - Results from the Open Call: How Citizens can participate in solar energy research?

http://doi.org/10.5281/zenodo.4452416 ^34^

This project contains the following underlying data:

- Answers to the online survey “Call for ideas_answers online_survey.xlsx”
- Notes from secretary groups of World Cafe and other meetings: “notes_MMLs_GRECO_2019.pdf”

Data are available under the terms of the Creative Commons Attribution 4.0 International license (CC-BY 4.0).
