# Supplementary material for: Promoting citizen science in the energy sector: Generation Solar, an open database of small-scale solar photovoltaic installations
Source: Open Res Eur. 2021 May 21;1:21. Originally published 2021 Mar 24. [Version 2] doi: 10.12688/openreseurope.13069.2 (PMC10445918; doi:10.12688/openreseurope.13069.2)
Supplement: Peer review history

# Peer review: Reviewer response for version 2

**DOI:** 10.21956/openreseurope.14791.r26948

**Published:** 2021-06-11

**Authors:** Wilfried van Sark

**Affiliations:** 1 Copernicus Institute of Sustainable Development, Utrecht University, Utrecht, The Netherlands

Thank you very much for answering to my concerns and remarks. I have no further comments.

Is the case presented with sufficient detail to be useful for teaching or other practitioners?

Partly

Is the work clearly and accurately presented and does it cite the current literature?

Partly

If applicable, is the statistical analysis and its interpretation appropriate?

No

Are all the source data underlying the results available to ensure full reproducibility?

Yes

Are the conclusions drawn adequately supported by the results?

Partly

Is the background of the case’s history and progression described in sufficient detail?

Partly

Reviewer Expertise:

Photovoltaics performance analysis, next generation PV design, integration of PV in the electricity system.

I confirm that I have read this submission and believe that I have an appropriate level of expertise to confirm that it is of an acceptable scientific standard.

# Peer review: Reviewer response for version 2

**DOI:** 10.21956/openreseurope.14791.r26949

**Published:** 2021-05-25

**Authors:** Destenie Nock

**Affiliations:** 1 Engineering and Public Policy Department, Carnegie Mellon University, Pittsburgh, PA, USA; 2 Civil and Environmental Engineering Department, Carnegie Mellon University, Pittsburgh, PA, USA

I think the authors have done well with addressing my comments. It is a very nice paper.

Is the case presented with sufficient detail to be useful for teaching or other practitioners?

Partly

Is the work clearly and accurately presented and does it cite the current literature?

No

If applicable, is the statistical analysis and its interpretation appropriate?

Yes

Are all the source data underlying the results available to ensure full reproducibility?

Yes

Are the conclusions drawn adequately supported by the results?

No

Is the background of the case’s history and progression described in sufficient detail?

Partly

Reviewer Expertise:

I am the director of the Energy, Equity, and Sustainability Group at Carnegie Mellon University. My work lies at he intersection of energy systems modeling, generation investment planning, and stakeholder decision analysis. In previous work I have led a stakeholder engagement in Ghana, and I have led an analysis of Northern Ireland's renewable energy deployment policies.

I confirm that I have read this submission and believe that I have an appropriate level of expertise to confirm that it is of an acceptable scientific standard.

# Peer review: Reviewer response for version 1

**DOI:** 10.21956/openreseurope.14139.r26633

**Published:** 2021-04-21

**Authors:** Wilfried van Sark

**Affiliations:** 1 Copernicus Institute of Sustainable Development, Utrecht University, Utrecht, The Netherlands

The authors describe the process to achieve a open data base especially targeted at small sized residential PV systems. This is an outcome of a process to define a suitable project focusing on citizen science. 

 While the process described to define such a project, it may be biased by the scientists involved in the brainstorming activities. It is unclear who those scientists were, or rather, their expertise and research field. Also, a strong dissemination campaign was undertaken to support a call for participation, with a result of 60 participants. This is a rather small amount. 

 Within the PV community as well as on various national levels, the need for proper registration of PV systems and especially their contribution to the renewable electricity generation is identified. Larger systems typically are required to report their energy yield in order to receive some kind of financial support from governments. Smaller systems not. 

 The change towards decentralised power systems requires knowledge about performance of those systems. This is important for utilities and network operators, but also for PV system owners, who typically do not know if their system is performing as promised by the installer.

 The paper does not recognise the work of Leloux ^1^ and Killinger ^2^, partly based on databases such as pvoutput.org and sonnenertrag.eu. Those databases include the metadata of systems with power generation data.

 What is the reason for the 1.1. km spatial resolution, other than privacy? Why not 1 or 2 km, for example.

 In addition to Reference 18, an analysis by Tsafarakis *et al.* ^3^ provides more details.

Is the case presented with sufficient detail to be useful for teaching or other practitioners?

Partly

Is the work clearly and accurately presented and does it cite the current literature?

Partly

If applicable, is the statistical analysis and its interpretation appropriate?

No

Are all the source data underlying the results available to ensure full reproducibility?

Yes

Are the conclusions drawn adequately supported by the results?

Partly

Is the background of the case’s history and progression described in sufficient detail?

Partly

Reviewer Expertise:

Photovoltaics performance analysis, next generation PV design, integration of PV in the electricity system.

I confirm that I have read this submission and believe that I have an appropriate level of expertise to confirm that it is of an acceptable scientific standard, however I have significant reservations, as outlined above.

## References

[1] : Performance to Peers (P2P): A benchmark approach to fault detections applied to photovoltaic system fleets. *Solar Energy* .2020;202: 10.1016/j.solener.2020.03.015 522-539 10.1016/j.solener.2020.03.015

[2] : On the search for representative characteristics of PV systems: Data collection and analysis of PV system azimuth, tilt, capacity, yield and shading. *Solar Energy* .2018;173: 10.1016/j.solener.2018.08.051 1087-1106 10.1016/j.solener.2018.08.051

[3] : Three years experience in a Dutch public awareness campaign on photovoltaic system performance. *IET Renewable Power Generation* .2017;11(10) : 10.1049/iet-rpg.2016.1037 1229-1233 10.1049/iet-rpg.2016.1037

# Response

**Published:** 2021-05-11

**Authors:** Carlos del Canizo

**Affiliations:** Universidad Politécnica de Madrid, Spain

We thank the reviewer for his valuable comments. *Comment #1: While the process described to define such a project, it may be biased by the scientists involved in the brainstorming activities. It is unclear who those scientists were, or rather, their expertise and research field. Also, a strong dissemination campaign was undertaken to support a call for participation, with a result of 60 participants. This is a rather small amount.*

 Unfortunately, we did not collect info on the professional background of the people answering the online survey. We do know it from the world cafés: energy, materials, physics,… We agree that the responses to the survey in step 1 will be biased by the scientists involved in the brainstorming activities. Clearly, the higher the number of experts and the variety of their origin and area of expertise, the better. But our point is in having as a starting point inputs from those who will benefit from the citizen engagement in their research, more than in guaranteeing that the survey is comprehensive and covers all potentially relevant topics. The dissemination campaign was devoted to the second step, that of the participation in the online hackathon. Taking into account that the participation meant the commitment to develop a project, alone or in a group, along a whole week, we think that reaching 60 people from 15 countries is a result of our efforts to reach public beyond what we are used to in our research projects... It is true that “strong” is an ambiguous word here… We have rephrased the text in section 3.2: “a call for participation was launched (Figure 1), and disseminated with the support of flyers, a newsletter, press releases, and a videoclip, translated to several languages, throughout different communication channels: GRECO website and social networks, local newspapers and magazines, networks of the GRECO partners.”

*Comment #2: Within the PV community as well as on various national levels, the need for proper registration of PV systems and especially their contribution to the renewable electricity generation is identified. Larger systems typically are required to report their energy yield in order to receive some kind of financial support from governments. Smaller systems not.  The change towards decentralised power systems requires knowledge about performance of those systems. This is important for utilities and network operators, but also for PV system owners, who typically do not know if their system is performing as promised by the installer.* We share the view expressed by the reviewer, and hope that Generation Solar can grow to deliver meaningful contributions to this knowledge.

*Comment #3: The paper does not recognise the work of Leloux and Killinger, partly based on databases such as pvoutput.org and sonnenertrag.eu. Those databases include the metadata of systems with power generation data*.

 We thank the reviewer for providing the references, which have been included in the paper. At the end of section 4, the text now says: “In this respect, Generation Solar can contribute to give support and continuation to works such as those of [Killinger et al], [Leloux et al], guaranteeing an open an easy access to its database.” 

*Comment #4: What is the reason for the 1.1. km spatial resolution, other than privacy? Why not 1 or 2 km, for exampl*e.

 This comes from the fact the way the app finds an equilibrium between privacy and location of the installation, the database takes only two decimals in the latitude and longitude coordinates, and discarding the rest of the decimals

*Comment #5: In addition to Reference 18, an analysis by Tsafarakis et al. provides more details*.

 Thank you for providing the reference of Tsafarakis et al, which updates and complements the findings of van Sark et al., and has been included in the introduction: “Efforts have been made to incorporate this kind of practices in the energy sector, covering aspects such as the evaluation of transition scenarios [Höfer et al], the engagement in energy efficiency and energy demand management [Cappa et al] [Filippo et al], the support in the performance assessment of photovoltaic systems [Tsafarakis et al] and the mapping of existing PV installations into OpenStreetMap [Stowell et al].”

# Peer review: Reviewer response for version 1

**DOI:** 10.21956/openreseurope.14139.r26625

**Published:** 2021-04-08

**Authors:** Destenie Nock

**Affiliations:** 1 Engineering and Public Policy Department, Carnegie Mellon University, Pittsburgh, PA, USA; 2 Civil and Environmental Engineering Department, Carnegie Mellon University, Pittsburgh, PA, USA

This case study details a citizen science engagement project for PV solar in Europe. The authors use surveys and face-to-face meetings to engage with professionals and a hack-a-thon type event to engage with a broader public. They use the hack-a-thon to gather citizen science ideas about solar projects and then present the final outcome of a PV generation database.

 Is the background of the case’s history describing in sufficient detail?

 I believe the contest used in section 3.2. is not described in enough detail. What was the goal of the working plan? What do you mean by strong dissemination? How is this like a hackathon?

 Is the work clearly and accurately presented and does it cite the current literature?

 I find this approach title (“Citizen Science for Citizen Science”) confusing. It seems redundant. I think the phrasing you used above “responsible citizen science” makes more sense.

 In the case study the authors state that citizen science experiences in the energy sector are scarce, and then goes to cite two papers. I think the comment about citizen science in the medical, astronomy, and other fields is distracting, because there is a wealth of citizen science initiatives in the energy sector. It would be better to provide citizen science approaches for different parts of the energy sector (generation, efficiency, renewables, etc.). I believe the work could also benefit from some citations about citizen science from a broader global context. This would aid in helping the energy community see possible learnings and opportunities for methods sharing across the globe. I offer possible citations below, but this is by no means exhaustive. One of the citations below points out that there are over 300 papers that investigate both energy efficiency and citizen science, so I am sure the authors will be able to find more for PV programs.

- Höfer, Tim, and Reinhard Madlener. "A participatory stakeholder process for evaluating sustainable energy transition scenarios." ^1^
- Pocock, M. J., Roy, H. E., August, T., Kuria, A., Barasa, F., Bett, J., ... & Trevelyan, R. (2019). Developing the global potential of citizen science: Assessing opportunities that benefit people, society and the environment in East Africa. ^2^
- Baker, Erin, et al. "Who is marginalized in energy justice? Amplifying community leader perspectives of energy transitions in Ghana."]ref-3].
- Leach, M., & Fairhead, J. (2002). Manners of contestation:“citizen science” and “indigenous knowledge” in West Africa and the Caribbean ^4^.
- Cappa, Francesco, et al. "Nudging and citizen science: The effectiveness of feedback in energy-demand management." ^5^.
- Filippo, Daniela De, et al. "Scientometric analysis of research in energy efficiency and citizen science through projects and publications." ^6^

If applicable, is the statistical analysis and its interpretation appropriate?

 N/A

 Are all the source data underlying the results available to ensure full reproducibility?

 Yes

 Are the conclusions drawn adequately supported by the results?

 One of the challenges I see in this paper is the survey participants in step 1 of the citizen science approach in the paper. The main questions presented in this section were: 1. How can citizens collaborate in our research? 2. What are the main barriers towards the integration of PV in society? 3. What does our research give in return to citizens? Yet, the brainstorming activities were conducted with 60 researchers, and the survey was delivered through academic channels. I would have expected the researchers to involved some members of the public who may be interested in the PV sector but are not directly involved in researching or deploying this technology. I understand that the public was involved in the hack-a-thon, but more detail should be given as to how the professional survey aided in the hack-a-thon. Also I expected to see some key takeaways from the survey.

 In section 3.4 the authors claim that the open model will aid in energy modeling. I am not seeing the connection. I think energy modelling is such a broad term that the authors need to comment on what type of open energy model they are hoping to aid. Based on section 4 it seems like the focus is on generation expansion modeling. Also I think for renewable energy modeling researchers need estimates for how much sun is in an area (this work might benefit from references to NREL PV Watts model, or Google’s Rooftop Solar Project).

 The conclusions section would benefit from a discussion about the value of this approach. How did the results here differ from other citizen science approaches, and how was the project enhanced by using the community engagement process? Also there should be some results from the professional survey, and then detail about how this survey fed into the citizen designs.

 Is the case presented with sufficient detail to be useful for other teachers and practitioners?

 I think that the case needs more detail on why the “Open database of rooftop solar PV installations”, was chosen as the winner. What set this project apart?

 Also there should be more detail about how the survey from the professionals connects to the final citizen science project. Right now it feels disjointed. Did the professionals get to help decide which project would move forward?

 Other notes:

 The text in Table 1 is off. In random places the words jump to the next line.

Is the case presented with sufficient detail to be useful for teaching or other practitioners?

Partly

Is the work clearly and accurately presented and does it cite the current literature?

No

If applicable, is the statistical analysis and its interpretation appropriate?

Yes

Are all the source data underlying the results available to ensure full reproducibility?

Yes

Are the conclusions drawn adequately supported by the results?

No

Is the background of the case’s history and progression described in sufficient detail?

Partly

Reviewer Expertise:

I am the director of the Energy, Equity, and Sustainability Group at Carnegie Mellon University. My work lies at he intersection of energy systems modeling, generation investment planning, and stakeholder decision analysis. In previous work I have led a stakeholder engagement in Ghana, and I have led an analysis of Northern Ireland's renewable energy deployment policies.

I confirm that I have read this submission and believe that I have an appropriate level of expertise to confirm that it is of an acceptable scientific standard, however I have significant reservations, as outlined above.

## References

[1] : A participatory stakeholder process for evaluating sustainable energy transition scenarios. *Energy Policy* .2020;139: 10.1016/j.enpol.2020.111277 10.1016/j.enpol.2020.111277

[2] : Developing the global potential of citizen science: Assessing opportunities that benefit people, society and the environment in East Africa. *Journal of Applied Ecology* .2019;56(2) : 10.1111/1365-2664.13279 274-281 10.1111/1365-2664.13279

[3] : Who is marginalized in energy justice? Amplifying community leader perspectives of energy transitions in Ghana. *Energy Research & Social Science* .2021;73: 10.1016/j.erss.2021.101933 10.1016/j.erss.2021.101933

[4] : Manners of contestation: “citizen science” and “indigenous knowledge” in West Africa and the Caribbean. *International Social Science Journal* .2002;54(173) : 10.1111/1468-2451.00383 299-311 10.1111/1468-2451.00383

[5] : Nudging and citizen science: The effectiveness of feedback in energy-demand management. *J Environ Manage* .2020;269: 10.1016/j.jenvman.2020.110759 110759 10.1016/j.jenvman.2020.110759 32425166

[6] : Scientometric Analysis of Research in Energy Efficiency and Citizen Science through Projects and Publications. *Sustainability* .2020;12(12) : 10.3390/su12125175 10.3390/su12125175

# Response

**Published:** 2021-05-11

**Authors:** Carlos del Canizo

**Affiliations:** Universidad Politécnica de Madrid, Spain

We thank the reviewer for her critical and constructive comments. We have tried to address her concerns in the new version of the paper. Find below our response to the specific comments:

*Comment #1: I believe the contest used in section 3.2. is not described in enough detail. What was the goal of the working plan? What do you mean by strong dissemination? How is this like a hackathon?*

 Section 3.2 have been rewritten to give more details on the second step of our initiative. The text now reads: “In this second phase, a call for participation was launched (Figure 1), and disseminated with the support of flyers, a newsletter, press releases, and a videoclip, translated to several languages, throughout different communication channels: GRECO website and social networks, local newspapers and magazines, networks of the GRECO partners. An online contest, inspired in a hackathon [Briscoe et al], was designed to motivate participants to develop a working plan and submit it for evaluation. In a hackathon, participants are brought virtually together to engage in brief, intensive collaborative work to solve a challenge. Although hackathons started linked to the field of software or IT development, they have more and more been used in all disciplines and proven very powerful for knowledge exchange and broader problem framing [Pe-Than et al], [Ghouila et al]. The contest finally gathered around 60 registered participants from 15 different countries, ran for an entire week and made use of the open source e-learning software Chamilo. Having as starting point the responses gathered in step 1, the participants, individually or in teams, had an entire week to propose an initiative in citizen science for the PV field, conveying a clear and feasible idea, together with the methodology and the tools to develop it. 

*Comment #2: I find this approach title (“Citizen Science for Citizen Science”) confusing. It seems redundant. I think the phrasing you used above “responsible citizen science” makes more sense.*

 With the expression “Citizen Science for Citizen Science”, even if it sounds redundant, we wanted to emphasize that as we see clearly the concept of Citizen Science for Health, Citizen Science for Energy… (in general, Citizen Science for a specific discipline), this “Citizen Science for…” can be applied to the Citizen Science itself, in the sense of engaging citizens in the co-creation and design of the initiative. This may be in the full Citizen Science concept, but it does not always happen. Anyway, we have removed the expression from the paper to avoid confusion.

*Comment #3: In the case study the authors state that citizen science experiences in the energy sector are scarce, and then goes to cite two papers. I think the comment about citizen science in the medical, astronomy, and other fields is distracting, because there is a wealth of citizen science initiatives in the energy sector. It would be better to provide citizen science approaches for different parts of the energy sector (generation, efficiency, renewables, etc.). I believe the work could also benefit from some citations about citizen science from a broader global context. This would aid in helping the energy community see possible learnings and opportunities for methods sharing across the globe. I offer possible citations below, but this is by no means exhaustive. One of the citations below points out that there are over 300 papers that investigate both energy efficiency and citizen science, so I am sure the authors will be able to find more for PV programs.*

 We thank the reviewer for suggesting to broaden the focus and reference citizen science initiatives in energy-related aspects, and offering some very interesting references. We have included some of them in the new version of the paper, and we have also incorporated some specific of the PV field. The paragraph now reads: “Efforts have been made to incorporate this kind of practices in the energy sector, covering aspects such as the evaluation of transition scenarios [Höfer et al], the engagement in energy efficiency and energy demand management [Cappa et al] [de Filippo et al], the support in the performance assessment of photovoltaic systems [Tsafarakis et al] and the mapping of existing PV installations into OpenStreetMap [Stowel et al]. It is clear that the urgent energy transition in which we are immersed, with the relevance of renewable energies, will only be possible through the concerted action of all stakeholders, putting into practice the benefits of a “quadruple helix of innovation” [Carayannis et al][Sanderik et al][Sauermanna et al], and going beyond social acceptance by reinforcing the participation in decision-making processes [Komentantoba et al] [Baker et al].“   

*Comment #4:  One of the challenges I see in this paper is the survey participants in step 1 of the citizen science approach in the paper. The main questions presented in this section were: 1. How can citizens collaborate in our research? 2. What are the main barriers towards the integration of PV in society? 3. What does our research give in return to citizens? Yet, the brainstorming activities were conducted with 60 researchers, and the survey was delivered through academic channels. I would have expected the researchers to involved some members of the public who may be interested in the PV sector but are not directly involved in researching or deploying this technology. I understand that the public was involved in the hack-a-thon, but more detail should be given as to how the professional survey aided in the hack-a-thon. Also I expected to see some key takeaways from the survey.*

 Step 1 was actually designed to include citizens as well, but it just so happened that, at the end, the majority of the responses (not all) came from researchers and professionals in the PV sector. Anyway, the focus here in our opinion is to consider that a citizen science initiative will only succeed if the researchers understand that it responds to a research question they are interested in, and realize that the participation of citizens indeed allows to address it with guarantees. We have made explicit this idea in the text by adding in section 3.1 the following text: “In order to launch a citizen science initiative with real value for the advancement of professional science, academic researchers must be included in the design phase, with the double objective of identifying relevant research questions and raising awareness of the support that citizens can provide to address them. This is especially valuable in engineering fields, less represented than biomedical and biological disciplines in public engagement actions.” The input from the interested public may be implicitly considered as the researchers themselves are not isolated and receive input from their professional and personal environments. Also, the questions in the survey were defined to broaden the perspective and make the respondents think beyond their specific research topic. This has been specified in the text: “For that, face-to-face brainstorming activities were carried out in several sessions in different countries to explore ideas from PV-related personnel around three questions, that were formulated to help the respondents think beyond their specific topics of expertise”. On the other hand, the text has been changed to explain that this professional survey was a starting point offered to the hack-a-thon participants to get inspired with the ideas. The last paragraph in section 3.1 now reads: “A 9-page document summarizing all responses collected (around 100, see underlying data) was compiled and is openly available in Zenodo [Cristóbal et al]. It gathers a handful of ideas that were offered as a starting inspiring point to the participants in step 2.”. This idea is also mentioned again in the modified text in section 3.2.: “Having as starting point the responses gathered in step 1, the participants, individually or in teams, had an entire week to propose an initiative in citizen science for the PV field…”

*Comment #5:  In section 3.4 the authors claim that the open model will aid in energy modeling. I am not seeing the connection.* *I think energy modelling is such a broad term that the authors need to comment on what type of open energy model they are hoping to aid. Based on section 4 it seems like the focus is on generation expansion modeling.* *Also I think for renewable energy modeling researchers need estimates for how much sun is in an area (this work might benefit from references to NREL PV Watts model, or Google’s Rooftop Solar Project).*

 The remark is very pertinent, and a clarification on what we mean by energy modelling is included in the last paragraph of section 3.3.: “The winning proposal aimed at providing support to scientists that model future energy systems with high renewable penetration by creating a collaborative database under an open license regarding the main characteristics of PV installations around the world, fed with the information provided by citizens.”. The mention to the energy modelling in the conclusions has also been modified to clarify this point: “Generation Solar is a Citizen Science Initiative in the energy field that takes advantage of the participation of citizens to build an open database of distributed solar PV installations, to give input to researchers working on future energy systems simulating different energy supply scenarios and predicting production from PV plants.” In this contribution we do not tackle the way that the “energy modellers” can use the data and elaborate on it; for sure, if they want to estimate the energy generated by the PV installations, they will need to complement the Generation Solar database with data on radiation and temperature. Today there are many tools to facilitate the calculation, the reviewer mentions a couple of online resources that can help on that, and many energy modellers have their own, with different degrees of sophistication.

*Comment #6:* *The conclusions section would benefit from a discussion about the value of this approach. How did the results here differ from other citizen science approaches, and how was the project enhanced by using the community engagement process?* *Also there should be some results from the professional survey, and then detail about how this survey fed into the citizen designs.*

 We tackle some of these questions in section 4. Besides, the conclusion section has been enriched with a paragraph tackling the aspects that the reviewer is pointing at: “The value of Generation Solar is not restricted to the usefulness of the database itself, for which the engagement of citizens is crucial due to the decentralized nature of PV installations; it also comes from the design process, that may serve as a guide for the development of other Citizen Science initiatives. Conceived to build a “responsible” citizen science initiative, it gives emphasis to participatory and co-creation aspects, to make sure that the initiative responds to a real need in the research field, and at the same time meets the expectations of citizens.”

*Comment #7: I think that the case needs* *more detail on why the “Open database of rooftop solar PV installations”, was chosen as the winner. What set this project apart? Also there should be more detail about how the survey from the professionals connects to the final citizen science project* *Right now it feels disjointed. Did the professionals get to help decide which project would move forward?*

 The winner scored the highest in the application of the assessment criteria in Table 1. This is now specified in section 3.3: “The highest score and hence the major prize…”. Also, the last paragraph in section 3.3 was rephrased to give more details about why the winning proposal was chosen, and a mention to the connection with the survey from professionals in step 1: “The winning proposal aimed at providing support to scientists that model future energy systems with high renewable penetration by creating a collaborative database under an open license regarding the main characteristics of PV rooftop installations around the world, fed with the information provided by citizens. In this way, it addressed some of the ideas coming from the survey in step 1 and summarized in [14]: for example, “Identifying locations where solar energy could replace other sources”, “Providing their experience, especially those that have installed a PV system at home”, “Sharing data from their solar devices”, “Send data of photovoltaic installations”, “[get to know] how solar energy will fulfil the energy needs in their daily life”, etc.”

*Comment #8: The text in Table 1 is off. In random places the words jump to the next line.* We have reformatted the table, and hopefully everything is Ok now.
